# Comparative study of endophytic bacterial strains from non-host crops for enhancing plant growth and managing early blight in tomato

**DOI:** 10.3389/fmicb.2024.1487653

**Published:** 2024-11-06

**Authors:** Aditya Kukreti, Chethana Bangi Siddabasappa, Prasannakumar Muthakapalli Krishnareddy, Pramesh Devanna, Yashavanth Basavapatna Subbanna, Manjunatha Channappa, Namburi Karunakar Reddy, Abeer Hashem, Mashail Fahad Alsayed, Elsayed Fathi Abd_Allah

**Affiliations:** ^1^Department of Plant Pathology, University of Agricultural Sciences, GKVK, Bengaluru, India; ^2^Insect Bacteriology Laboratory, ICAR-National Bureau of Agricultural Insect Resources, Bengaluru, India; ^3^Rice Pathology Laboratory, ARS, Gangavathi, India; ^4^National Academy of Agricultural Research Management, Hyderabad, India; ^5^Botany and Microbiology Department, College of Science, King Saud University, Riyadh, Saudi Arabia; ^6^Plant Production Department, College of Food and Agricultural Sciences, King Saud University, Riyadh, Saudi Arabia

**Keywords:** endophytes, tomato, *Bacillus* spp., *Alternaria solani*, early blight, plant growth promotion

## Abstract

*Bacillus pseudomycoides*, *Paenibacillus polymyxa*, and *B. velezensis* are potent bacterial endophytes, which typically exhibit host-specific interactions. However, comparative studies of these endophytes *in vitro* and *in planta* in non-host crops are lacking. Therefore, in this study, we evaluated the potential of endophytes *B. pseudomycoides* strain HP3d, *P. polymyxa* strain PGSS1, *B. velezensis* strain A6, and P42, isolated from various crop ecosystems in promoting plant growth and inducing systemic resistance against early blight disease in tomato. *In vitro*, endophytes exhibited 44.44–55.56% and 37.50–87.50% inhibition of *Alternaria solani* in dual culture and volatilome bioassay, respectively. In the glasshouse, individual and combined applications via seed treatment (ST), seedling dip (SD), and foliar spray (FS) significantly enhanced shoot growth (23.63–57.61%), root growth (43.27–118.23%), number of leaves (77.52–93.58%), number of shoots (33.42–45.28%) and root dry matter (42.17–43.86%), reducing early blight (PDI) by 70.95–76.12% compared to uninoculated control. Enzymatic activities, including such as polyphenol oxidase (30–40 fold), peroxidase (65.00–75.00 fold), superoxide dismutase (34.20–37.20 fold) and phenylalanine ammonia-lyase (44.44–45.56 fold) were elevated post-inoculation in endophytes treated tomato plants challenged with *A. solani* compared to control treated only with *A. solani* and declined after the fifth day. The total chlorophyll content declined from the 0th to the 10th day, but endophyte treated plants exhibited lesser reductions (2.03–2.09) than uninoculated control. Field trials confirmed the glasshouse findings, showing reduced early blight and improved growth parameters in tomato where the ST + SD + FS combination emerged as the most effective treatment for all endophytes showing 1.06–1.88 fold increase in fruit yield per plant and 28.92–32.52% decrease in PDI compared to untreated control. Thus, the study highlights the broad-spectrum potential of these strains in promoting plant growth and controlling early blight in tomato, demonstrating non-host specificity. These endophytes offer eco-friendly alternatives to chemical pesticides, supporting sustainable agriculture. Their success in field trials suggests the potential for commercialization and large-scale use across diverse crops and pave the way for further interdisciplinary research to optimize their application in integrated pest management strategies.

## 1 Introduction

Tomato (*Solanum lycopersicum* L.) is a high-demand crop with widespread use and nutritional benefits in both fresh and processed markets. India is a major tomato producer with 355.48 million tonnes of production, cultivating an area of 28.44 million hectares (Ministry of Agriculture and Farmers Welfare, 2022–2023). However, a variety of fungal, bacterial, viral, and nematode diseases have hampered commercial tomato production worldwide (Adhikari et al., 2017). Of the economically important diseases of tomato, early blight caused by *Alternaria solani* (Ellis and Martin, 1882) has become one of the most destructive disease with yield losses of 35–78% worldwide (Karacic et al., 2024) and up to 80 percent in India. The disease primarily affects tomato plant leaves, stems, and fruits resulting in severe defoliation, decreased productivity, and low fruit quality (Chaerani et al., 2007). Multiple strategies such as resistant tomato varieties along with cultural, physical and chemical approaches, have been used worldwide to manage tomato diseases. However, these methods have been found less efficient in controlling *A. solani*. Chemical pesticides remain an effective method for controlling pathogen and minimizing yield losses (Karacic et al., 2024). Currently, bacterial antagonists, specifically endophytes serving as biological control agents (BCAs), have garnered significant interest as a viable alternative for managing early blight (Pane and Zaccardelli, 2015; Shoaib et al., 2019) due to concerns associated with chemical fungicides, including the emergence of fungicide resistance, the buildup of residues in fruits, the loss of helpful phylloplane and soil bacteria, and environmental pollution (Chowdappa et al., 2013). Among BCAs, *Bacillus* stands out as a highly promising biocontrol owing to its Gram-positive nature, ability to generate endospores, and remarkable resilience against heat and desiccation (Kukreti et al., 2023; Ankitha et al., 2023). These qualities make it exceptionally suitable for both storage and field deployment. Given that pathogenic microorganisms and endophytic bacteria share the same niche within plants, inoculating plants with endophytic bacteria is an effective biological method for controlling pathogens (Wu et al., 2021).

Emerging as prominent endophytes for biocontrol, *Bacillus velezensis*, *Paenibacillus* sp., and *Bacillus pseudomycoides* exhibit significant potential. *Bacillus velezensis*, a widely distributed Gram-positive bacterium named in 2005 (Ruiz-Garcia et al., 2005), stimulates plant root growth through nutrient uptake and secretion of secondary metabolites like indole-3-acetic acid. It also combats fungal growth with polyketides, lipopeptide antibiotics, and enzymes (Kim et al., 2017). *Paenibacillus* sp., another endophyte, excels in producing hydrolyzing enzymes, forming protective root biofilms, and emitting pathogen-inhibiting volatile compounds (Timmusk and Wagner, 1999; El-Deeb et al., 2013). On the other hand, *B. pseudomycoides*, a facultative anaerobic gram-positive bacterium, showcases biocontrol prowess through strains like strain NBRC 101232, effectively countering *Ralstonia solanacearum* (Yanti et al., 2018). These dynamic endophytes collectively contribute promising avenues for enhanced biocontrol strategies.

In our previous investigations, *B. velezensis* strains A6 and P42 (isolated from pomegranate and rice respectively) were extensively examined, revealing numerous potent bioactive secondary metabolites, including antimicrobial peptides (AMPs) like fengycin, iturin, bacillomycin, and surfactin (Amruta et al., 2018). These discoveries were facilitated through a diverse array of techniques such as whole-cell sequencing, whole-cell protein profiling, thin-layer chromatography, infra-red spectroscopy, nuclear magnetic resonance, gas chromatography, and electro spray liquid chromatography which affirmed these strains biocontrol efficacy (Prasanna et al., 2021). The fundamental mechanisms underlying their biocontrol activities involve a range of strategies, including the production of siderophores, hydrogen cyanide (HCN), both volatile and non-volatile organic chemicals, and lytic enzymes (Kukreti et al., 2023). Moreover, they induce systemic resistance (ISR) by activating key enzymes in the phenylpropanoid pathway, such as phenylalanine ammonia-lyase, peroxidase, polyphenol oxidase, and superoxide dismutase (Shabanamol et al., 2017). Beyond disease management, these agents also contribute to plant growth promotion by enhancing nutrient acquisition and modulating phytohormone levels (Rana et al., 2011; Manivannan et al., 2012).

There have been conflicting reports regarding endophyte original host species specificity. According to several studies, endophytes can only encourage the growth of their original host (Long et al., 2008). Conversely, endophytes have been associated with the growth of a variety of plant hosts (Ma et al., 2011; Sessitsch et al., 2005) indicating that this aspect is not yet definitively understood. Non-host endophytes present significant advantages due to their ability to colonize a variety of plant species and adapt to different environmental conditions. Unlike host-specific strains, which are often limited to their native hosts, non-host endophytes exhibit greater ecological adaptability. This allows them to form beneficial relationships with a broader range of plants, enhancing traits like stress tolerance, disease resistance, and growth across multiple crops. Research has demonstrated that endophytes can colonize non-host plants and provide habitat-specific stress tolerance to their non-hosts, indicating that symbiotic interactions may be crucial in enhancing plant stress resilience (Byregowda et al., 2022). Their capacity to provide habitat-specific stress tolerance makes them particularly valuable in regions with harsh climates or poor soil conditions. Additionally, their adaptability means they can be used in diverse agricultural systems, offering a sustainable and flexible alternative to traditional host-specific strains (Choudhary et al., 2023). By utilizing non-host endophytes, farmers could improve crop resilience, boost yields, and reduce dependency on chemical inputs, making these endophytes ideal for developing broad-spectrum biofertilizers and biopesticides that work across a range of environments. Thus focusing on non-host endophytes can be advantageous and can open a new realm of biocontrol tactics.

Moreover, while plant-beneficial endophytic bacteria hold great promise as biofertilizers and biopesticides, their practical effectiveness in the field has often been limited (Afzal et al., 2019). Thus, in the present study, the antagonistic effects of *B. velezensis* strain A6 and P42, *P. polymyxa* PGSS-1, and *B. pseudomycoides* HP3d were evaluated *in vitro* against *A. solani* via dual culture and volatilome assay. Under glasshouse conditions, these endophytes were assessed for their ability to promote plant growth and exhibit antagonistic activity. Additionally, the involvement of enzymes such as PPO, POD, PAL, and SOD in the induced systemic response of tomato plants against *A. solani* infection was analyzed. Field trials were also conducted to explore the real world effect of these endophytes to highlight their potential as valuable tools in crop management.

## 2 Materials and methods

### 2.1 Bacterial strains, plant material, and pathogen isolation

The characterized non-pathogenic four endophytic bacterial strains such as *B. velezenesis* strain P42 (from pomegranate, GenBank 16 s rRNA accession number KC692168), *B. velezenesis* strain A6 (from rice, GenBank 16 s rRNA accession number MSXZ01000332), *Paenibacillus polymyxa* strain PGSS-1 (from ragi) and *B. pseudomycoides* strain HP3d (from rice, GenBank 16 s rRNA accession number MH465502) and a reference strain *Pseudomonas fluorescens* were procured from the Bacteriology Laboratory, Department of Plant Pathology, UAS, GKVK, Bengaluru, Karnataka, India and maintained at 4°C and stored at −20°C with 20% glycerol for further study. Seeds of tomato variety Arka Vikas (susceptible to early blight) were obtained from the Indian Institute of Horticultural Research (IIHR), Bengaluru, Karnataka, India. Pathogen was isolated from the tomato leaves showing typical symptoms of early blight by using potato dextrose agar (PDA) medium and identified as *A. solani* according to Ellis and Martin (1882).

### 2.2 *In vitro* bioassay

#### 2.2.1 Dual culture

Evaluation of bacterial endophytes against *A. solani* was conducted by dual culture technique. The 7 day old pathogen grown on PDA and 48 h old bacterial cultures of P42, HP3D, PGSS 1, A6 and *P. fluorescens* (positive control) grown on nutrient agar (NA) were used for the test. The actively growing mycelial disks of the *A. solani* (5 mm) were positioned on one side of a Petri dish with PDA media, while bacterial endophytes were streaked on the opposite side, with three replications maintained (Latha et al., 2009). Inoculated plates were incubated at 28 ± 1°C. In the control setup, the pathogen was inoculated without any bacterial treatment. The results of the performance of endophytic bacteria against test fungi were recorded after the complete growth of the pathogen was seen in control plates. The colony diameter was measured and compared with control plates. The percentage inhibition of the pathogen compared to the control was determined using the formula given by Vincent (1947).


$$\mathrm{PIRG}=\frac{R1-R2}{R1}\times 100.$$

Where, PIRG—percent inhibition of radial growth. R1—radial growth of test fungi in control plate. R2—radial growth of test fungi in dual culture with endophyte.

#### 2.2.2 Volatiolome assay

The experiment aimed to evaluate the inhibition of mycelial growth of *A. solani* through the emission of antifungal volatile organic compounds (VOCs) by the bacterial endophyte and *P. fluorescens* (positive control). A 5 mm agar plug from the edge of an actively growing seven-day-old colony of *A. solani* was inoculated on the base plate of the Petri plate containing 15 mL PDA. Subsequently, the base plate of another petri plate containing 15 mL NA was streaked with potential bacterial endophyte, and the two plates were sealed immediately, using a double layer of parafilm to make a closed chamber plate. Plates were incubated at 28 ± 1°C until maximum growth was observed in control plates (Rouissi et al., 2013). The percent inhibition was calculated by using the formula of Vincent (1947).

### 2.3 Assessment of endophytes in the glasshouse

#### 2.3.1 Plant growth

The experiment involved culturing four endophytic strains (P42, A6, HP3d, and PGSS-1) along with positive control (*P. fluorescens*) (David et al., 2018), in NA broth for 48 h at a temperature of 28 ± 2°C. Following centrifugation at 6,000 × g for 5 min, the samples were rinsed and resuspended in sterile water to reach a 10^9^ cfu/mL concentration (Chowdappa et al., 2013). These strain’s PGP activity was then assessed in a glasshouse setting by two factorial completely randomized design (CRD). For each endophyte application, six distinct modes of application were experimented *viz.*, seed treatment (ST, T1), seedling dip (SD, T2), ST combined with foliar spray (FS) (T3), SD combined with FS (T4), ST combined with SD (T5), and a combination of ST, SD, and FS (T6) in a completely randomized block design; and each treatment was replicated three times. Uninoculated seeds served as the negative control.

Two sets, designated as Group A and Group B, were formed using tomato seeds. Group A was further subdivided based on bacterization with five different endophytes. Group B, which was non-inoculated and surface-sterilized, was used exclusively for SD treatment and as a negative control. For surface sterilization in ST (T1), group A tomato seeds underwent a two-minute treatment with NaClO (Chowdappa et al., 2013), followed by three complete washes using sterile water. Following, the seeds were subjected to treatment with 10 mL/kg of fresh endophytic cultures (10^9^ cfu/mL), grown in nutrient broth with 0.2% CMC (5 mL/gm of seed as a sticker). After treatment, the seeds were incubated for 24 h, shade-dried, and then sown in sterile soil in plastic trays kept in a glasshouse for 21 days. For the SD treatment (T2), pots were sterilized and filled with a mixture of soil, cocopeat, and FYM in a 1:1:1 ratio. The field soil and cocopeat were autoclaved twice for 45 min at 121°C, with a 24-h interval between sessions. A portion of the seeds from group B (uninoculated) was planted and later transferred into half of these pots 21 days after sowing (DAS) after dipping in a 48-h-old endophyte culture diluted in water (10 mL/L) for 30 min. The remaining half of the pots received seedlings from group A, which had been dipped in a 48-h-old endophyte culture, constituting treatment T5. FS of 24 h old endophytes suspension (1 × 10^9^ CFU/ml) diluted in water @ 10 mL/L was done for part of ST (T3), SD (T4), and ST + SD (T6) seedlings, 10 days after transplanting (DAT). The examination of the parameters *viz.*, stem height, root length and number of leaves for tomato were recorded at 20 DAT. The weight of roots and shoots, both fresh and dry, was also recorded post-harvest by drying to a constant weight in an oven at 60°C for 3 days (Latha et al., 2009).

#### 2.3.2 Lesion size and early blight intensity

To investigate the antagonistic effects of endophytes against the early blight pathogen, plants 45 days old from each of the six treatments were inoculated with *A. solani* (2 × 10^7^spores/mL suspension). The affected plants were placed in a humidity chamber to allow normal disease development, by maintaining optimal conditions with 90% humidity and a stable temperature of 26 ± 4°C, and symptom progression was monitored 10 days post-inoculation (Latha et al., 2009). Plants inoculated only with the *A. solani* were used as positive controls, whereas those sprayed with sterilized water served as negative controls. Symptoms were assessed based on lesion size (measured with a ruler), and the severity of early blight was determined by calculating the ratio of lesion height in endophyte-treated plants to lesion height in positive control plants (Shoaib et al., 2019). This value was expressed as a percentage 15 days post-inoculation, when the plants were 60 days old.

#### 2.3.3 Enzymatic assays

To study enzymatic activities within the above glasshouse setup, mature leaves were sampled on the 0th, 3rd, 5th, 7th and 10th days after the inoculation challenge with *A. solani*. One gram of leaf tissue was homogenized using liquid nitrogen with a pre-cooled pestle and mortar, and the resulting powder was ground in 3 mL of a solution containing 0.1 M sodium phosphate buffer with polyvinylpyrrolidone (PVP) and ethylenediaminetetraacetic acid (EDTA) at pH 7.0. The homogenate was then centrifuged at 10,000 rpm for 20 min at 4°C, and the resulting supernatant was utilized as a crude enzyme extract for subsequent assays, including the evaluation of polyphenol oxidase (PPO), peroxidase (POD), phenyl ammonia lyase (PAL) and superoxide dismutase (SOD) activity. PPO activity was assessed following the method outlined by Manoranjan and Mishra (1976), with changes in absorbance at 420 nm recorded every 30 s for 3 min, expressed as a change in absorbance/min/g of fresh tissue. For POD activity Saroop et al. (2002) method was followed and monitoring absorbance increases at 420 nm at 30-s intervals for 3 min, also expressed as a change in absorbance/min/g of fresh tissue. SOD activity was determined by its ability to inhibit the photochemical reduction of nitro blue tetrazolium (NBT) following Beauchamp and Fridovich (1971), reported in units/g/fresh weight. PAL activity was assayed as described by Campos et al. (2004), with the assay mixture read at 290 nm and expressed as a change in absorbance/min/g of fresh tissue. All steps in the extract preparation were conducted at 4°C. Each enzyme assay included three replicates (each replicate comprising five plants) and two spectrophotometric readings per replicate.

#### 2.3.4 Chlorophyll estimation

Chlorophyll content assessment was performed on the 0th, 5th, and 10th day following the challenge inoculation with *A. solani*, according to the protocol outlined by Hiscox and Israelstam (1979). Chlorophyll a, b and total chlorophyll content were computed through the formulas given by Arnon (1949).

### 2.4 *In planta* assessment

#### 2.4.1 Preparation of endophytes inoculum

For ST, 24 h culture broth of endophytes (1 × 10^9^ cfu/mL) was prepared, incorporating Tween 20 at a concentration of 10 mL/L of water and 0.2% CMC (Latha et al., 2009) at 5 mL/g. For SD, the 24 h culture endophytes broth was mixed with 0.2% CMC and diluted to 10 mL/L of water and for the FS, the 24 h endophytes broth was diluted to 10 mL/L of water with 0.1% Tween 20.

#### 2.4.2 Application of endophytes preparation *in planta*

Four endophytes P42, HP3d, PGSS 1, and A6, including *P. fluorescens* as a positive control, uninoculated plants as a negative control, and a recommended fungicide Captan 50% WP (DPPQ, India), were evaluated against *A. solani* in a sick plot at the University of Agricultural Sciences, GKVK, Bengaluru, Karnataka, India, during the summer of 2021. Tomato seeds were sown in sterile trays filled with cocopeat and 21 days seedlings were transplanted into experimental plots, with 10 plants per row. The tomato plants were placed in 5 × 4 m^2^ plots with a spacing of 60 × 30 cm. Agricultural practices, including irrigation and fertilization, were applied timely in the field based on the recommendation of Kumar et al. (2017). Evaluation against early blight was conducted under natural conditions. The experiment utilized a randomized block design (RBD) with 22 treatments, each replicated thrice. The treatments with endophytes were applied as mentioned in Table 1. For ST, tomato seeds underwent surface sterilization by immersing them in a 2% NaClO solution for 2 min, followed by three thorough rinses with sterile distilled water. The seeds were then air-dried under a sterile air stream and were immersed in ST preparation. The treated seeds were air-dried and sown in the trays. For SD, 30-day-old tomato seedlings were dipped in SD preparation for 1 h before being transplanted into the field. FS involved spraying FS preparation onto the plants at 15-day intervals after the onset of symptoms during the cropping season, with three sprays administered.

**Table 1:** List of treatments evaluated against early blight of tomato in field conditions.

Sl. No.	Treatments*
T1	E1 T1 – Seed treatment @ 10 mL/kg + CI
T2	E1 T2 – Seedling dip @ 10 mL/L + CI
T3	E1 T3 – T1 + T2 + CI
T4	E1 T4 – T1 + T2 + Foliar Spray + CI
T5	E2 T1 – Seed treatment @ 10 mL/kg + CI
T6	E2 T2 – Seedling dip @ 10 mL/L + CI
T7	E2 T3 – T1 + T2 + CI
T8	E2 T4 – T1 + T2 + Foliar Spray + CI
T9	E3 T1 – Seed treatment @ 10 mL/kg + CI
T10	E3 T2 – Seedling dip @ 10 mL/L + CI
T11	E3 T3 – T1 + T2 + CI
T12	E3 T4 – T1 + T2 + Foliar Spray + CI
T13	E4 T1 – Seed treatment @ 10 mL/kg + CI
T14	E4 T2 – Seedling dip @ 10 mL/L + CI
T15	E4 T3 – T1 + T2 + CI
T16	E4 T4 – T1 + T2 + Foliar Spray + CI
T17	PF T1 – Seed treatment @ 10 mL/kg + CI
T18	PF T2 – Seedling dip @ 10 mL/L + CI
T19	PF T3 – T1 + T2 + CI
T20	PF T4 – T1 + T2 + Foliar Spray+ CI
T21	Fungicide Captan 50% WP
T22	Control

*E1- P42, E2- HP3d, E3- PGSS 1, E4- A6; PF, Pseudomonas fluorescence; CI, challenge inoculation.

Observations on the various growth parameters, including shoot length, number of branches per plant, number of trusses per plant, number of fruits per truss, number of fruits per plant, equatorial diameter of fruit, average fruit weight, and fruit yield per plant, were recorded at 100 days after transplanting (DAT). Additionally, the fruit yield per hectare was noted after harvesting. A10 plants were selected randomly from each plot and disease scoring following each treatment spray was conducted using a five-point scale (0–5) (Khan et al., 2023) and percent disease index (PDI) was calculated using formula of Wheeler (1969).

### 2.5 Statistical analysis

The normality of error distribution was assessed using the Shapiro–Wilk test and the homogeneity of error variance was checked using the Bartlett test. When the assumptions of normality and homogeneity of variance were met, ANOVA followed by the Tukey test was used to compare all treatments, with a significance level set at 5%. Data were analyzed using two-way ANOVA for the glasshouse conditions and one-way ANOVA for the field conditions. The least significant difference (LSD) test was performed to separate the group means when ANOVAs were significant at *p* < 0.05. Prior to statistical analysis, all percentage data were subjected to angular transformation to stabilize variances. Means were compared using Tukey’s Honest Significance Difference (HSD) test. Graphs were generated using the ggplot2 package in R software. PCA was carried out using FactoMineR (Husson et al., 2020) and Factoextra (Kassambara and Mundt, 2020) packages in R.

## 3 Results

### 3.1 *In vitro* bioassay

#### 3.1.1 Dual culture

**Figure 1:**
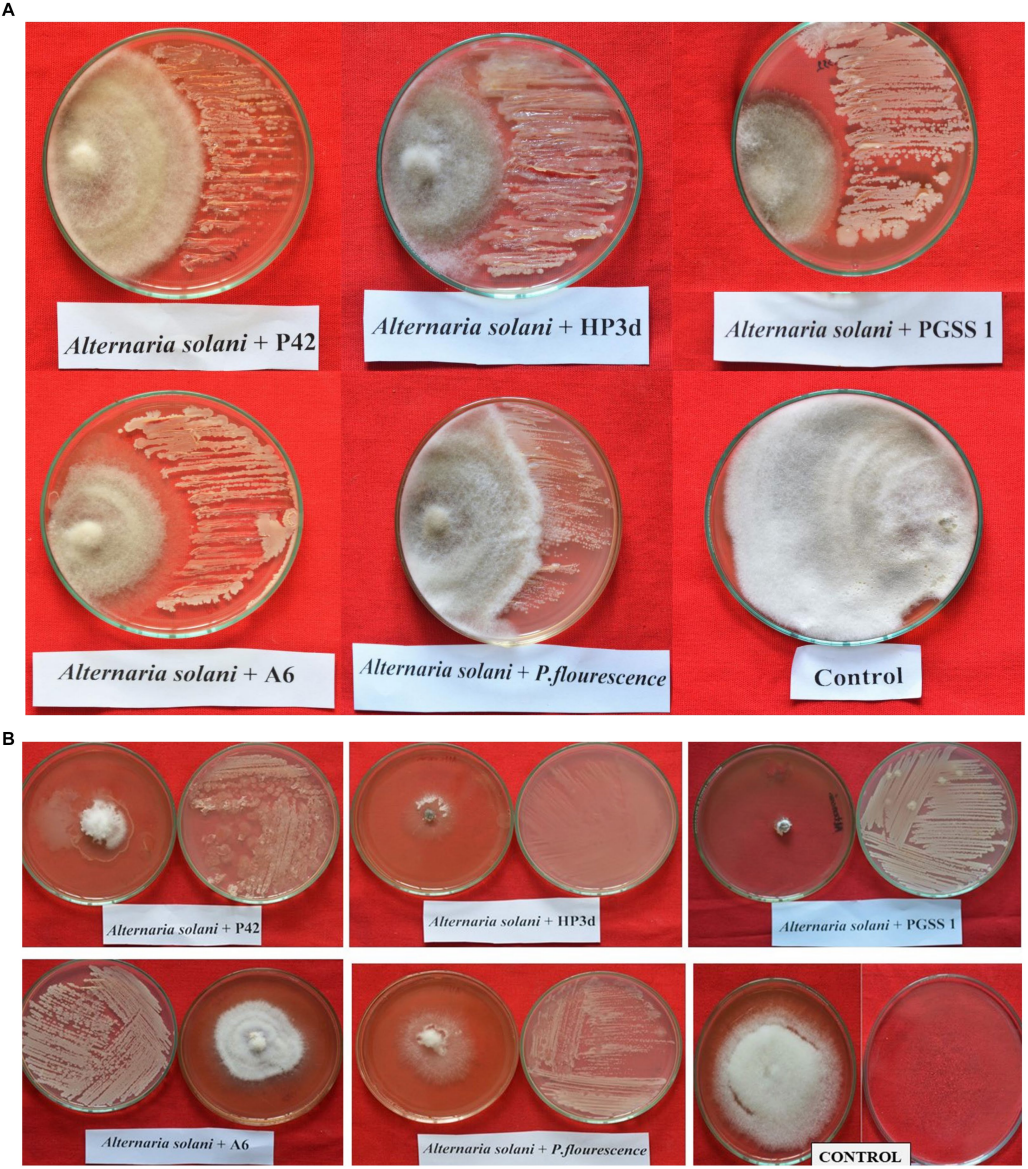
*In vitro* evaluation of the antagonistic potential of bacterial endophytic strains against *Alternaria solani* through **(A)** dual culture technique **(B)** Volatilome bioassay.

All four bacterial endophytes inhibited *A. solani* growth, with percent inhibition over negative control ranging from 44.44 to 55.56% (Table 2; Figure 1A). HP3d, PGSS1 and A6 were the most effective, each achieving a maximum inhibition of 55.56%. This was followed by P42 with 44.44% inhibition. All treatments showed significant inhibition compared to *P. fluorescens* which exhibited 33.33% inhibition.

**Table 2:** Evaluation of the antagonistic effect of endophytic bacterial strains on *Alternaria solani* using dual culture and volatilome bioassays.

Bacterial endophytes	Dual culture		Volatilome assay	
	RMG (mm)*	% Inhibition**	ARMG (mm)*	% Inhibition**
*Bacillus velezensis* P42	50^c^	44.44^b^	25^d^	68.75^b^
*Bacillus pseudomycoides* HP3d	40^d^	55.56^a^	10^e^	87.50^a^
*Paenibacillus polymyxa* PGSS1	40^d^	55.56^a^	10^e^	87.50^a^
*Bacillus velezensis* A6	40^d^	55.56^a^	50^b^	37.50^d^
*Pseudomonas fluorescens* (positive control)	60^b^	33.33^c^	40^c^	50.00^c^
Control (negative control)	90^a^	–	80^a^	–

*ARMG-Average radial mycelial growth; **% Inhibition-% inhibition of mycelial growth over control; ***Means followed by same letters are not significantly different according to Duncan’s multiple range test (DMRT) at 5% level.

#### 3.1.2 Volatiolome assay

All bacterial endophytes tested effectively inhibited the growth of *A. solani*, with a percent inhibition over control ranging from 37.50 to 87.50 (Table 2; Figure 1B). Among the four bacterial endophytes, HP3d and PGSS1 recorded a maximum percent inhibition of 87.50, followed by P42 (68.75), and were significantly superior over *P. fluorescens* (50.00). Interestingly, A6 recorded a minimum of 37.50.

### 3.2 Assessment of endophytes in the glasshouse

#### 3.2.1 Plant growth

**Figure 2:**
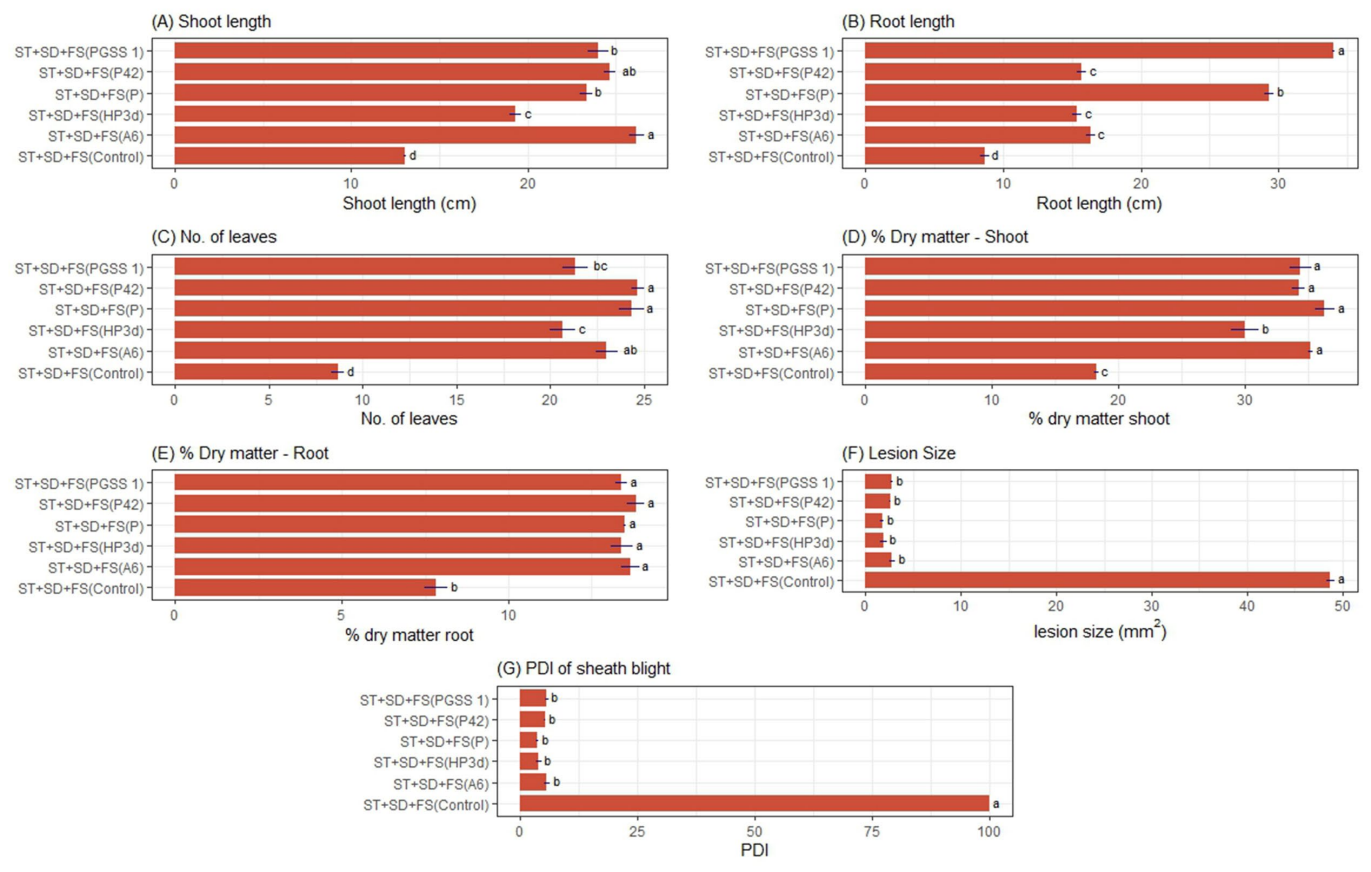
Effect of ST + SD + FS of bacterial endophytes on tomato under glasshouse conditions. The bar graph illustrates **(A)** shoot length, **(B)** root length, **(C)** number of leaves, **(D)** % shoot dry matter, **(E)** %root dry matter, **(F)** Lesion size (mm^2^), and **(G)** %disease severity index (PDI) of early blight. The values shown are the averages from three independent experiments, with standard errors represented by error bars. Different letters on the bars indicate significant differences according to DMRT at *p* < 0.05.

A two-way ANOVA experiment was conducted to evaluate the impact of two independent variables (endophytes and mode of treatment) on growth parameters. The results indicated that bacterial endophytes significantly enhanced tomato growth, in terms of increased root length, number of leaves, shoot length, and the percentage of shoot and root dry matter over the negative control. The shoot length in different endophyte treatments revealed significant variations. The percent increases in shoot length were 40.96, 23.63, 54.11, and 57.61% in plants treated with P42, HP3d, PGSS-1, and A6 compared to the negative control, whereas on comparing to the *P. fluorescens*, only PGSS-1 and A6 showed an increase of 2.87 and 5.22%. Among the modes of treatments, the endophytes applied through ST + SD + FS recorded the highest shoot length of 21.76 cm, whereas ST alone recorded the lowest of 14.77 cm (Supplementary Table S1; Figure 2A). In the case of root length, P42, HP3d, PGSS1, and A6 showed an increase of approximately 43.27, 45.26, 118.23, and 64.92%, respectively, over the negative control; however, all four endophytes exhibited a lower percent increase in root length compared to *P. fluorescens*, which demonstrated a 143.38% increase. Additionally, ST + SD + FS recorded the highest root length of 19.88 cm, whereas ST recorded the lowest of 10.59 cm (Supplementary Table S2; Figure 2B). For the number of leaves per plant, compared to the control, all treatments resulted in a significant increase in the number of leaves. P42 exhibited the highest increase (95.23%), followed by A6 (92.20%), PGSS1 (82.15%), and HP3d (77.52%) compared to the negative control. When comparing the treatments to *P. fluorescens*, which showed an increase of about 93.58% over negative control, P42 still showed a slight increase of about 0.89%, whereas A6, PGSS1, and HP3d had lower numbers of leaves than *P. fluorescens*. In all endophytes, ST + SD + FS recorded the highest number of leaves per plant, 20.45, whereas ST recorded the lowest 11.95 (Supplementary Table S3; Figure 2C).

The shoot dry matter content was measured across different endophyte treatments and revealed a significant percent increase in dry matter content compared to the negative control with P42, HP3d, PGSS1, and A6 showing a 40.92, 33.42, 45.28, and 43.33% increase. None of the endophytes exhibited a higher percentage increase than *P. fluorescens* (52.92%). Among modes of treatment, ST + SD + FS recorded the highest shoot dry matter of 31.35%, followed by SD + FS (28.61%) and SD (27.64%), with ST (19.65%) being the lowest (Supplementary Table S4; Figure 2D). Regarding root dry matter content, P42, HP3d, PGSS1, and A6 showed a 43.73, 42.17, 43.86, and 43.86% increase over the negative control, respectively, and in terms of percent increase over *P. fluorescens*, P42 showed a 0.59% increase, and both PGSS1 and A6 showed a 0.67% increase. Pairwise comparisons among the treatments revealed no significant differences. ST + SD + FS of endophytes recorded the highest root dry matter of 12.60%, which was on par with ST + FS of 12.19%, whereas ST recorded the lowest of 9.68 (Supplementary Table S5; Figure 2E).

#### 3.2.2 Lesion size and early blight intensity

An ANOVA experiment was performed to evaluate the combined effects of two independent variables, endophytes, and treatment mode, on lesion size and early blight intensity. A decrease in lesion size compared to *P. fluorescens* and the negative control was recorded in P42 (11.51 and 74.80%), HP3d (16.78 and 76.12%), and PGSS1 (4.97 and 72.75%), respectively. A6 exhibited a 70.95% decrease over negative control but did not show compared to *P. fluorescens*. ST + SD + FS (10.07 mm^2^), SD + FS (11.17 mm^2^) and SD (12.21 mm^2^) exhibited a minimum and ST (38.14 mm^2^) maximum lesion size (Supplementary Table S6; Figure 2F). Subsequently, based on the lesion size, the severity of early blight was assessed, which showed that P42 (30.03), HP3d (28.48), PGSS1 (32.48), and A6 (34.60) displayed reduced PDIs in contrast to both *P. fluorescens* (34.18) and the control (100.00) on treatment via ST + SD + FS (20.66), SD + FS (23.01) and SD (25.33) (Supplementary Table S7; Figure 2G).

#### 3.2.3 Enzymatic assays

**Figure 3:**
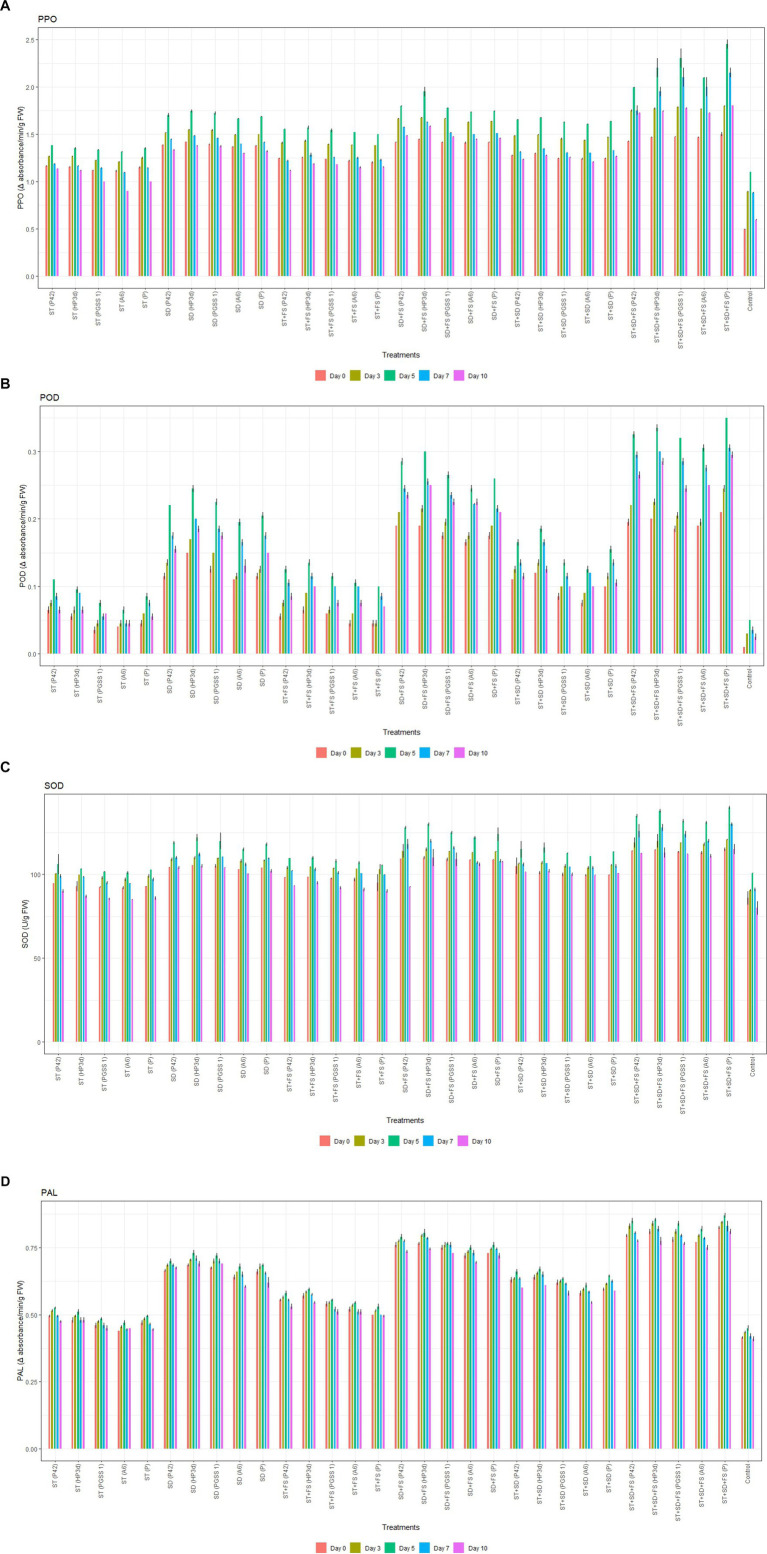
Effect of various bacterial endophyte treatments on **(A)** PPO (change in absorbance min^−1^ g^−1^), **(B)** POD (change in absorbance min^−1^ g^−1^ FW), **(C)** SOD (U g^−1^ FW), and **(D)** PAL (change in absorbance min^−1^ g^−1^ FW) activity following inoculation with *A. solani* in tomato plants under glasshouse conditions. The data represent the mean values from three replications, with standard errors indicated by error bars.

The enzyme activity was significantly stimulated in all 30 treatments under pathogenic stress when compared to the negative control (without endophyte inoculation), and enzyme levels showed a gradual rise until the fifth day post-inoculation with *A. solani* followed by a subsequent decline. On fifth day, the highest activity of PPO was recorded in the treatment, ST + SD + FS with PGSS1 (2.30 min^−1^ g^−1^) followed by HP3d (2.20 min^−1^ g^−1^), A6 (2.10 min^−1^ g^−1^) and P42 (2.00 min^−1^ g^−1^) over uninoculated control (1.10 min^−1^ g^−1^) thus exhibiting an increase of 30–40 fold over uninoculated control whereas showed lower activity compared to *P. fluorescens* (2.50 min^−1^ g^−1^) (Figure 3A). For POD, the highest activity was recorded in the treatment ST + SD + FS with HP3d (0.34 min^−1^ g^−1^), followed by ST + SD + FS with P42 (0.33 min^−1^ g^−1^), PGSS1 (0.32 min^−1^ g^−1^) and A6 (0.31 min^−1^ g^−1^), on the 5th day after challenged inoculation with *A. solani* and thus showed 65.00–75.00 fold increase over uninoculated control (0.05 min^−1^ g^−1^). All the endophytes showed low POD activity in comparison to *P. fluorescens* (Figure 3B). Likewise, SOD activity was significantly amplified in HP3d (138 Ug^−1^ FW) and P42 (135 Ug^−1^ FW), which were on par with each other and with *P. fluorescens* (140 Ug^−1^ FW) on the 5th day after challenged inoculation with *A. solani* and exhibited 34.20–37.20 fold increase over uninoculated control (Figure 3C). For PAL, ST + SD + FS with HP3d (0.86 min^−1^ g ^−1^) and P42 (0.85 min^−1^ g ^−1^) were on par with each other and with *P. fluorescens* (0.87 min^−1^ g ^−1^) on 5th day after challenged inoculation with *A. solani* and showed a 44.44–45.56 fold increase over uninoculated control (Figure 3D).

#### 3.2.4 Chlorophyll estimation

**Figure 4:**
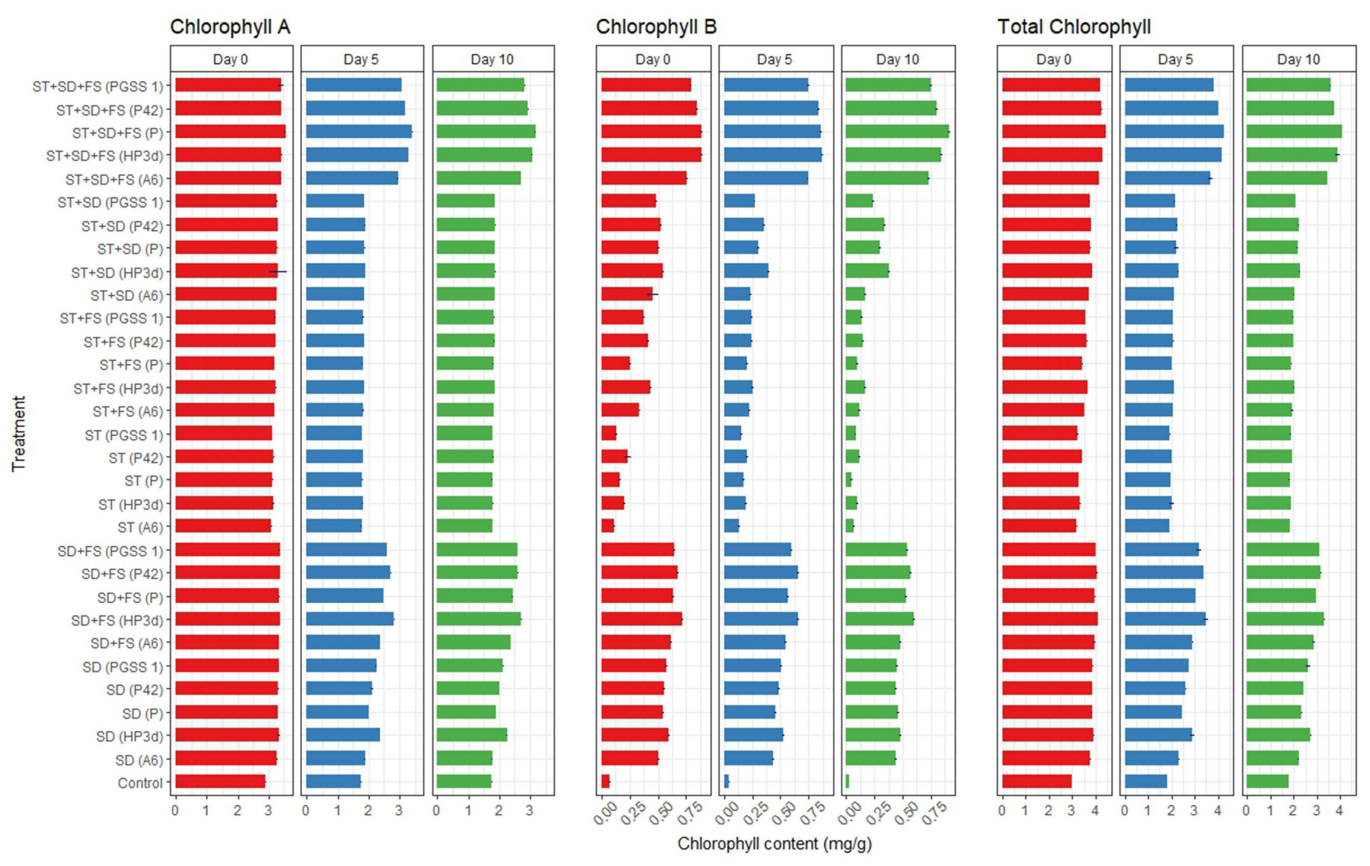
Effect of various bacterial endophyte treatments on chlorophyll content (mg/g) measured on 0th, 5th, and 10th day following inoculation with *A. solani* in tomato plants under glasshouse conditions. The data represent the mean values from three independent experiments, with standard errors indicated by error bars.

Among 30 treatments and on the 5th day, endophytes inoculated early blight infected plants recorded significant decreased fold reduction in chlorophyll “a”, “b,” and total chlorophyll content ranged from 1.67–1.86, 15.00–17.50, and 2.03–2.09, respectively, compared to the uninoculated control however showed a comparable decrease to *P. fluorescens*. The concentration was higher on the 0th day, dropped on the 5th day, and was lowest on the 10th day. The highest chlorophyll concentration after 10 days was recorded in ST + SD + FS, while the lowest concentration was recorded in ST with endophytes (Figure 4).

### 3.3 Principle component analysis

**Figure 5:**
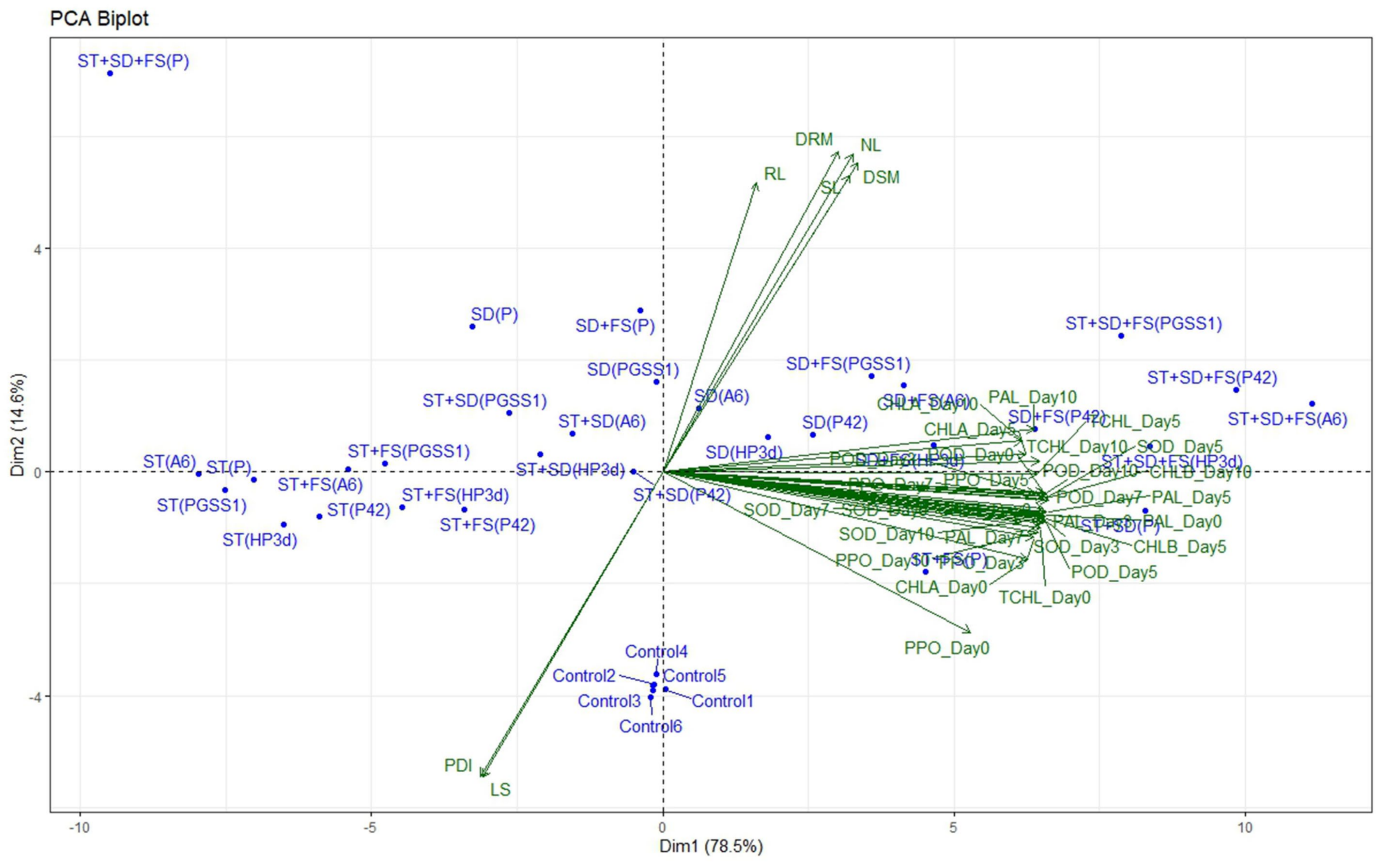
Principal component analysis (PCA) of the effect of endophytes individually and in different combinations on the plant growth parameters with percentage disease index in the glasshouse, represented by biplot. PDI, percentage disease index; SL, shoot length; RL, root length; NL, number of leaves; LS, lesion length; DSM, dry shoot matter; DRM, dry root matter.

The impact of individual endophytes and their combinations on plant growth, defense-related enzymes, and yield parameters in the glasshouse, as compared to the percentage disease index (PDI), was examined through principal component analysis (PCA). The principal components accounted for 93.11% of the total variance, as illustrated in Figure 5. Specifically, Component 1 (Dim1) contributed to 78.52% of the variance, while Principal Component 2 (Dim2) accounted for 14.59%. In the PCA biplot, it was noted that variables with narrow angles (less than 90 degrees) displayed a strong positive correlation and directly influenced each other. In contrast, the PDI and lesion length (LS) were positioned in a different quadrant, indicating a negative correlation with other variables. On the positive axis of Dim1, variables such as PPO, SOD, PAL, POD, stem length (SL), root length (RL), number of leaves (NL), dry shoot matter (DSM), dry root matter (DRM), total chlorophyll (TCHL), chlorophyll a (CHLA), and chlorophyll b (CHLB) had a significant impact. Meanwhile, LS and PDI primarily influenced Dim2 on the positive axis.

### 3.4 *In planta* assessment

**Figure 6:**
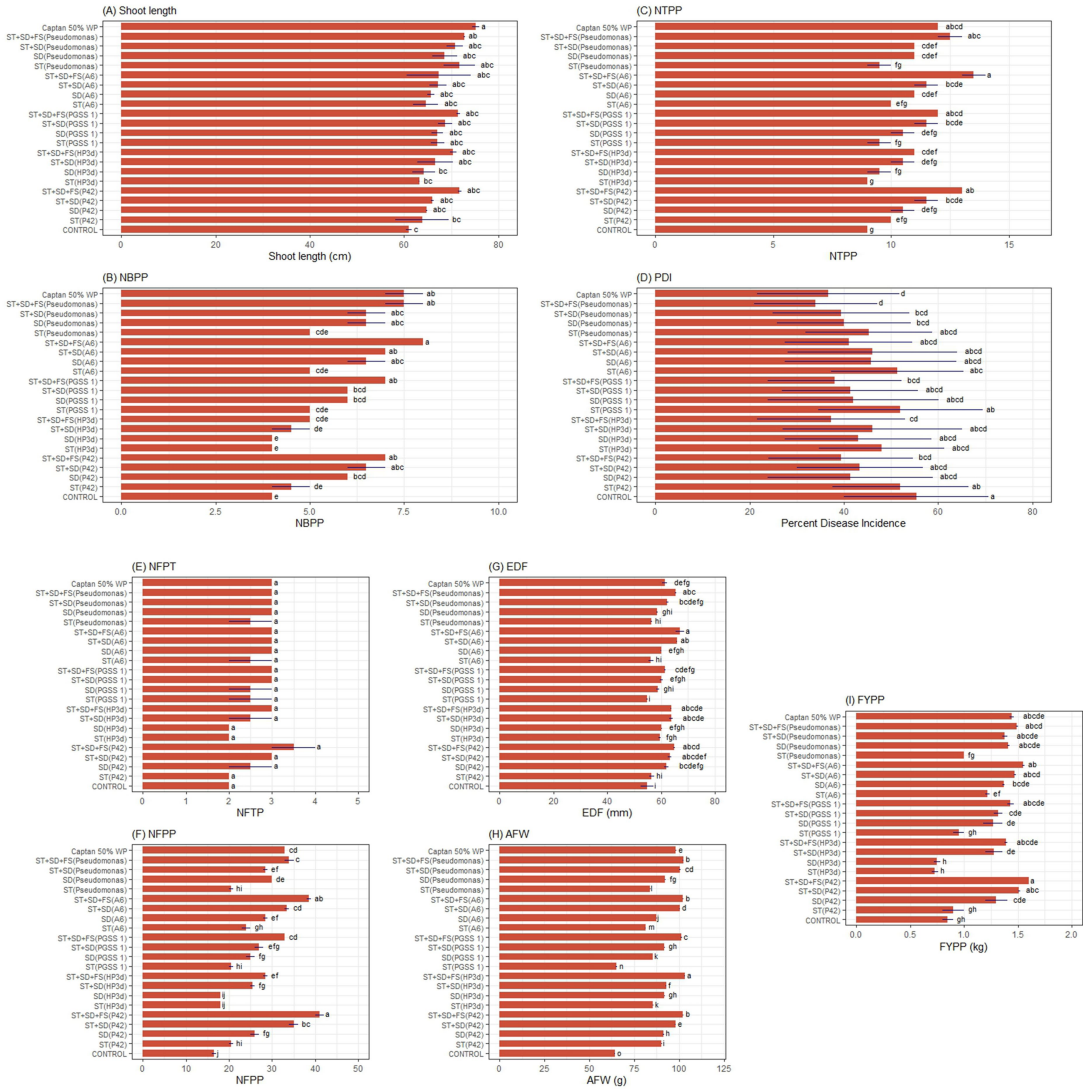
Effect of different endophyte treatments on the **(A)** shoot length (cm), **(B)** NBPP-no. of branches per plant, **(C)** NTPP-no. of trusses per plant, **(D)** PDI-Percent disease index, PDI-Percent disease index, **(E)** NFPT-no. of fruits per truss, **(F)** NFPP-no. of fruits per plant, **(G)** EDF-equatorial diameter of fruit, **(H)** AFW-average fruit weight, **(I)** FYPP-fruit yield per plant, of tomato under field conditions.The figures presented are the mean values of three independent experiments. Standard errors of the mean values are presented as bars. Letters on bars indicate DMRT at *p* < 0.05.

All endophytic treatments exhibited statistically significant higher shoot length, number of branches per plant (NBPP), number of trusses per plant (NTPP), number of fruits per plant (NFPP), equatorial diameter of fruit (EDF), average fruit weight (AFW) and fruit yield per plant (FYPP), compared to the uninoculated control. Endophyte treatments showed significantly higher mean shoot length exhibiting a 1.10–1.17 fold increase compared to the untreated control but resulted in relatively lower mean length compared to *P. fluorescens* and captan 50% WP (Figure 6A). For NBPP, all the treatments showed a 1–2 fold increase in NBPP, compared to the untreated control, and ST + SD + FS (A6) exhibited the highest mean values comparable to Captan 50% WP and ST + SD + FS (*P. fluorescens*). Also, treatments such as ST + SD + FS (PGSS 1), ST + SD + FS (P42), and ST + SD (PGSS 1) exhibited significant effectiveness over other treatments (Figure 6B). All the treatments significantly increased the NTPP by 1.11–1.50 fold over the untreated control, where ST + SD + FS (A6) showed the highest NTPP comparable to Captan 50% WP treatment and was insignificant to ST + SD + FS (P42), ST + SD + FS (PGSS 1) and ST + SD + FS (*P. fluorescens*) (Figure 6C). For PDI, only ST + SD + FS (PGSS 1), ST + SD + FS (HP3d), and ST + SD + FS (P42) showed significantly low PDI, i.e., 38.00, 37.33, and 39.33 over uninoculated control (55.33) and comparable to Captan 50% WP (36.67) and *P. fluorescens* (Figure 6D). No significant difference in NFPT was seen between any treatments (Figure 6E). Compared to the control, all treatments exhibit a significant increase in NFPP except ST (HP3d) and SD (HP3d), whereas ST + SD (P42), ST + SD + FS (PGSS 1) and ST + SD (A6) were comparable to *P. fluorescens* (ST + SD + FS) and Captan 50% WP (Figure 6F).

All the treatments significantly increased the EDF by 1.00–1.22 fold over the untreated control, where ST + SD + FS (A6) showed the highest EDF (1.22 fold over control) compared to Captan 50% WP (1.12 fold over control) but was comparable to ST + SD + FS (*P. fluorescens*) (1.19 fold over control) and was insignificant to treatments ST + SD (P42), ST + SD + FS (P42), ST + SD (HP3d), ST + SD + FS (HP3d), and ST + SD (A6) (Figure 6G). All the treatments significantly increased the AFW by 1.01–1.60 fold over the untreated control, where ST + SD + FS (HP3d) showed the significantly highest AFW (1.60 fold over control) compared to Captan 50% WP (1.12 fold over control) and ST + SD + FS (*P. fluorescens*) (1.59 fold over control). ST + SD + FS (A6) showed a 1.59 fold over control comparable to *P. fluorescens* (Figure 6H). Regarding FYPP, leaving SD (HP3d) and ST (HP3d), all the treatments showed a significant increase in the FYPP (1.06–1.88 fold increase) over the untreated control. ST + SD + FS (P42) and ST + SD + FS (A6) showed the highest FYPP, and was insignificant in comparison to ST + SD + FS (HP3d), ST + SD + FS (PGSS 1), Captan 50% WP and ST + SD + FS (*P. fluorescens*) which exhibited 1.64, 1.68, 1.69 m, and 1.75 increase fold in FYPP over uninoculated control (Figure 6I) (Supplementary Table S8).

## 4 Discussion

The mutualistic relationship between host plants and bacterial endophytes is crucial in terrestrial ecosystems as host plants provide shelter and protection to endophytes, which, in turn, enhance plant growth through nitrogen fixation, phosphorus enrichment, and synthesis of beneficial compounds (Wu et al., 2021); however, the specificity of endophyte-plant interactions is not clear. Many studies have documented that endophytic bacteria can enhance the growth of only plant species from which they have been isolated, but there are also reports of endophytic bacteria benefiting non-host plants (Afzal et al., 2019). In our earlier studies, strains A6 and P42 have been thoroughly analyzed, uncovering a wide range of bioactive secondary metabolites, particularly AMPs such as fengycin, iturin, bacillomycin, and surfactin (Amruta et al., 2018). These findings were confirmed using various advanced methods, including whole-cell sequencing, protein profiling, thin-layer chromatography, infrared spectroscopy, nuclear magnetic resonance, gas chromatography, and electro-spray liquid chromatography, which validated the biocontrol potential of these strains (Prasanna et al., 2021). Therefore, to explore the potential non-host specificity, adaptability, and bioefficacy of the four characterized endophytes, we evaluated their plant growth promotion, ISR activity, and antagonistic properties under both *in vitro* and *in vivo* conditions.

Endophytic bacteria are promising BCAs for controlling foliar phytopathogens, but to successfully identify their potency, it is crucial to use an appropriate *in vitro* evaluation system in the preliminary stages (Wang et al., 2019). Thus, to assess the biocontrol potential of endophytes, their efficacy against *A. solani* was examined through *in vitro* bioassay via dual culture and volatilome approach, where they showed inhibition ranging from 44.44–55.56% and 37.50–87.50% over negative control. HP3d and PGSS1 exhibited significantly higher effectiveness than *P. fluorescens*, making them the most effective among the four endophytes tested. The inhibition observed in the dual culture assay is likely due to the production of hydrolytic enzymes and antifungal metabolites that act directly on the fungal cell wall, disrupting it and thus impeding fungal radial expansion (Ruqiya et al., 2022). This inhibitory potential of antagonistic bacteria is crucial for their competitive advantages in environments, including spatial competition, nutrient utilization, and oxygen availability (Karim et al., 2018), allowing antagonistic bacteria to flourish and effectively suppress pathogen growth. In the volatilome bioassay, the inhibition was due to VOCs emitted by endophytic bacteria, which are highly significant as they play a critical role in inhibiting the growth and germination of plant pathogen spores and also offer a distinct advantage over diffusible antibiotics as they can disperse over considerable distances, establishing fungistatic microenvironments around communities of antagonists (Wang et al., 2019).

Enhancing host plant resilience to various stresses by promoting plant growth is a key defense strategy employed by plants against pathogen attacks. In the present study to assess the PGP activities of our endophytes, glasshouse studies were carried out where different tomato growth parameters, *viz.*, root length, number of leaves, shoot length, and the percentage of shoot and root dry matter showed a significant increase over uninoculated tomato seedlings (negative control) but were either comparable or less potent than positive control (*P. fluorescens*). Among the different methods of endophyte application, while their effectiveness varied depending on the growth parameter examined, treatments via ST + SD + FS, SD + ST and SD demonstrated the best results and thus were the most effective methods for the application of these endophytes in tomato plants. Among the endophytes, PGSS-1 and A6 were the most efficient in increasing the growth parameters but were less efficient in reducing the early blight of tomato compared to P42 and HP3d. The differences in the effectiveness of the bacteria in promoting growth and reducing disease can be attributed to several factors, where the possible reasons for these observations can be because PGSS-1 and A6 might have mechanisms more involved in promoting tomato growth, such as producing plant hormones (like auxins or gibberellins), solubilizing phosphates, or fixing nitrogen which can directly enhance plant growth parameters, on the other hand, P42 and HP3d might possess more robust antimicrobial properties, producing antibiotics, specific metabolites or enzymes that directly inhibit the *A. solani*, thereby reducing the early blight incidence more effectively (Xia et al., 2022). The same glasshouse study was used to understand the role of various enzymes potentially involved in reducing early blight incidence in plants and the activities of key defense enzymes, including PPO, POD, SOD, and PAL in tomato following challenge inoculation with *A. solani*. The enzyme activities were recorded to be elevated in all treatments compared to the negative control. The results corroborated earlier findings of the efficacy of P42 and HP3d, which demonstrated the smallest lesion size and lowest PDI, alongside the highest enzymatic activity. Among the treatment modes, ST + SD + FS, SD + FS, and SD were most effective in enhancing enzymatic activity in tomato. While defense enzyme activities also increased in non-bacterized tomato plants inoculated with *A. solani*, the activities were significantly higher in bacterized plants which could be attributed to the presence of a complex network of antioxidative defense systems to counter harmful reactive oxygen species (ROS), which include free radicals like OH^−^ and O^2−^, as well as non-radicals like H_2_O_2_ and O_2_, formed under biotic stress. The ROS scavenging mechanism involves the upregulation of enzymatic components such as including PPO, POD, SOD, and PAL, to reduce pathogen invasion (Awan et al., 2018; Huang et al., 2019). Also, infected tomato plants exhibited a significant reduction in photosynthetic pigments, specifically chlorophyll content, likely due to chlorophyll degradation associated with leaf blight symptoms (Xie et al., 2015). However, the reduction was more pronounced in uninoculated plants compared to those inoculated with endophytes. Furthermore, PCA showed a positive correlation between all the yield parameters of tomato and defense-related enzymes, whereas defense-related enzymes showed a negative correlation with PDI. These findings are consistent with those of Shoaib et al. (2019), who observed similar results in tomatoes treated with strains of *Bacillus subtilis* and *Pseudomonas maltophilia*. Thus, the study showed that in glasshouse conditions, there was an increase in PGP and disease reduction in tomato which infers to improved plant health and thus increased yield.

While numerous studies have assessed the effectiveness of endophytes *in vitro*, many of them do not translate into successful outcomes in field conditions. *In vitro* studies typically involve direct exposure of endophytes to pathogens on synthetic media, facilitating direct interaction between the endophyte and the pathogen. However, *in planta* evaluations occur under natural conditions, exposing endophytes to various factors that can influence their biological activity in the field (Prasannakumar et al., 2020). These factors include the ability of bacteria to colonize the plant, characteristics of the host plant, and environmental conditions. Key determinants encompass the plant’s age, genotype, geographical location, growth stage, and specific tissues examined. Climatic conditions also significantly impact the abundance and composition of endophytic bacteria. Soil type influences endophytic diversity, leading to variations in endophyte populations even within the same plant cultivar grown in different soils. Moreover, plants can selectively recruit endophytic bacteria in response to stress factors like the presence of phytopathogens. This dynamic selection process is closely regulated by the host plant to promote the growth and defense of beneficial bacteria (Afzal et al., 2019). In our present study, we found that in field trials in particular environmental conditions, all plots treated with endophytes exhibited a significantly higher number of branches per plant, number of trusses per plant, number of fruits per plant, equatorial diameter of fruit, average fruit weight and fruit yield per plant compared to the uninoculated control and were comparable to the effects of captan. The treatment combination of ST @10 mL/kg + SD@10 mL/L + FS @10 mL/L of water yielded the most effective results. The field trial data clearly show that all endophytic treatments led to statistically significant improvements in key growth and yield parameters compared to the uninoculated control. However, variability across treatments is evident. For example, treatments such as ST + SD + FS (P42) and ST + SD + FS (A6) achieved a 1.88-fold increase in fruit yield per plant (FYPP), while others like SD (HP3d) and ST (HP3d) showed much lower gains. This variability could be attributed to differences in how effectively each endophyte colonizes the plant, as well as their interactions with local soil and environmental factors. The inconsistent performance of HP3d treatments, particularly in FYPP and fruit weight (AFW), compared to more successful treatments suggests that certain endophytes may not be as robust across all growth conditions. In terms of practical agricultural implications, while the average improvements in shoot length (1.10–1.17 fold), number of branches, and fruit yield (1.06–1.88 fold) suggest strong potential for enhancing tomato productivity, it is important to note that these results were achieved under controlled conditions. The impact of environmental factors such as soil type, moisture, and temperature in real-world farming systems could lead to different outcomes. For example, while Captan 50% WP and *P. fluorescens* showed comparable or better results in some metrics (e.g., shoot length and fruit yield), endophytic treatments performed similarly or better in others, indicating room for improvement in optimizing their application. Inconsistencies, like the relatively lower effect of some HP3d treatments on fruit yield and shoot length, could result from less effective colonization or antagonism against the pathogen *A. solani* compared to other endophytes. Such variations underscore the need for a more detailed investigation into how environmental variables (e.g., temperature, soil composition) and endophyte-plant compatibility contribute to these outcomes. Further trials across different climatic conditions and crop systems will help refine these findings and better understand how to maximize the effectiveness of these biocontrol agents in diverse agricultural settings.

Comparing our findings with previous studies, we find strong alignment with past research that highlights the plant growth-promoting and disease suppressive ability of *Bacillus* and *Paenibacillus* strains. However, our study extends this understanding by demonstrating their potential in non-host crops in particular environmental conditions. Thus, utilizing specialized model systems in future studies will allow for deeper investigation into these complex interactions, helping to fine-tune the application of these endophytes to efficiently suppress pathogens like *A. solani* and sustainably increase tomato yields. Contradictory findings from earlier studies, where environmental factors or crop specificity limited efficacy, suggest that further research is essential to identify the specific plant and environmental factors that may influence the performance of these endophytes. While our study demonstrates the clear effectiveness of these endophytes in both laboratory and field trials, further validation across other crops and diverse environmental conditions is necessary to confirm their broad-spectrum efficacy.

The findings of this study present opportunities for translating these non-host endophytes, into bioformulations to enhance crop growth and disease resistance, offering an eco-friendly alternative to chemical pesticides. However, several challenges must be addressed to facilitate their widespread adoption in agricultural practices. Formulation is a critical aspect, as effective delivery methods must be developed to ensure that the endophytes can survive and thrive in diverse environmental conditions while reaching the target plants. Scalability poses another challenge; mass-producing these beneficial microorganisms in a cost-effective manner is essential for large-scale implementation. Moreover, the cost-effectiveness of using these bio-inoculants compared to traditional chemical treatments must be evaluated, as farmers are often hesitant to adopt new technologies unless they can demonstrate clear economic benefits. Addressing these challenges through targeted research and development will be crucial to harnessing the full potential of these non-host endophytes in sustainable agriculture.

## 5 Conclusion

In this study, four endophytic strains (P42, A6, HP3d, and PGSS1), isolated from various crops, were assessed using different application methods in both *in vitro* and *in vivo*, showing a non-host specific nature and broad-spectrum activity in tomato. While the *in vitro* evaluation displayed their effectiveness against *A. solani*, relying solely on the identification of biocontrol bacterial endophytes based on their performance on laboratory media tends to favor organisms functioning through antibiosis or hyperparasitism and potentially overlooking those that act through competition or ISR. Therefore, screening for antagonists in glasshouse conditions, where tomato plants were kept in autoclaved soil rather than agar, was conducted where they not only reduced early blight PDI through increased expression of various defense-related enzymes *viz.*, PPO, POD, PAL, and SOD but also enhanced PGP activities, regarding them as effective bacterial biocontrol agents in tomato. However, the relatively uniform environmental conditions in pot tests compared to field situations often lead to an overestimation of antagonistic and PGP properties, so a field study was conducted for these strains, where the treated tomato plants exhibited the same results as in pot tests, i.e., higher PGP and lower PDI, resulting in increased yield, thus highlighting the potency and wide adaptability of these endophytes in tomato ecosystem in particular environmental, glasshouse and field conditions. Further research to explore their potency in other crops and environmental conditions is needed and a study on biochemical, molecular, and genetic mechanisms involved can provide insights into their competency. Thus, future work on limitations of this study, i.e., the need for additional testing of these endophytes in different environmental conditions, crop species and climatic scenarios to guarantee the wider applicability of the findings; and exploring the interactions between these endophytes and various biotic and abiotic factors in diverse agricultural systems is essential. Conducting these thorough evaluations will help validate the efficacy and adaptability of these bio-inoculants across a range of farming practices. Additionally, developing application methods for large-scale field use, including formulation and delivery, could facilitate the utilization of these endophytes as efficient, cost-effective, and environmentally friendly bioformulations in the tomato ecosystem.

## Data Availability

The datasets presented in this study can be found in online repositories. The names of the repository/repositories and accession number(s) can be found in the article/Supplementary material.
