# Supplementary material for: Comparative study of endophytic bacterial strains from non-host crops for enhancing plant growth and managing early blight in tomato
Source: Front Microbiol. 2024 Nov 6;15:1487653. doi: 10.3389/fmicb.2024.1487653 (PMC11576455; doi:10.3389/fmicb.2024.1487653)
Supplement: Supplementary file 1 [file Table_1.DOCX]

**Supplementary table 1:** Effect of bacterial endophytes on shoot length (cm) of tomato under glasshouse conditions by two-way ANOVA

| **TREATMENTS** | **SHOOT LENGTH (cm)** | | | | | | |
| --- | --- | --- | --- | --- | --- | --- | --- |
|  | **P42** | **HP3d** | **PGSS-1** | **A6** | ***P.fluorescens*** | **Control** | **Mean** |
| **Seed treatment** | 14.33 | 13.17 | 17.00 | 16.33 | 15.43 | 12.33 | 14.77 |
| **Seedling dip** | 18.17 | 15.67 | 19.10 | 19.77 | 19.67 | 12.67 | 17.51 |
| **Seed treatment +foliar spray** | 14.50 | 13.93 | 17.83 | 17.50 | 16.83 | 12.50 | 15.52 |
| **Seedling dip+ foliar spray** | 21.00 | 17.83 | 21.83 | 23.00 | 21.50 | 13.07 | 19.71 |
| **Seed treatment + seedling dip** | 15.33 | 14.83 | 18.33 | 18.00 | 18.00 | 13.00 | 16.25 |
| **Seed treatment + seedling dip+ foliar spray** | 24.67 | 19.33 | 24.00 | 26.20 | 23.33 | 13.03 | 21.76 |
| **Mean** | 18.00 | 15.79 | 19.68 | 20.13 | 19.13 | 12.77 |  |
|  | **SEm**$\boldsymbol{\pm}$ | **CD at 1%** | **CD at 5%** |  |  |  |  |
| **Treatments (T)** | 0.18 | 0.69 | 0.52 |  |  |  |  |
| **Endophytes (E)** | 0.18 | 0.69 | 0.52 |  |  |  |  |
| **TXE** | 0.45 | 1.69 | 1.28 |  |  |  |  |

**Supplementary table 2:** Effect of bacterial endophytes on root length (cm) of tomato under glasshouse conditions by two-way ANOVA

| **TREATMENTS** | **ROOT LENGTH (cm)** | | | | | | |
| --- | --- | --- | --- | --- | --- | --- | --- |
|  | **P42** | **HP3d** | **PGSS-1** | **A6** | ***P.fluorescens*** | **Control** | **Mean** |
| **Seed treatment** | 9.13 | 9.43 | 11.00 | 11.60 | 15.03 | 7.37 | 10.59 |
| **Seedling dip** | 12.30 | 12.77 | 16.60 | 14.30 | 18.90 | 8.67 | 13.92 |
| **Seed treatment +foliar spray** | 10.67 | 10.80 | 13.60 | 12.00 | 17.17 | 8.27 | 12.09 |
| **Seedling dip+ foliar spray** | 13.77 | 13.89 | 20.33 | 15.50 | 24.33 | 8.90 | 16.12 |
| **Seed treatment + seedling dip** | 11.00 | 11.33 | 15.00 | 13.77 | 18.50 | 8.77 | 13.06 |
| **Seed treatment + seedling dip+ foliar spray** | 15.67 | 15.33 | 34.00 | 16.33 | 29.33 | 8.63 | 19.88 |
| **Mean** | 12.09 | 12.26 | 18.42 | 13.92 | 20.54 | 8.44 |  |
|  | **SEm**$\boldsymbol{\pm}$ | **CD at 1%** | **CD at 5%** |  |  |  |  |
| **Treatments (T)** | 0.11 | 0.42 | 0.32 |  |  |  |  |
| **Endophytes (E)** | 0.11 | 0.42 | 0.32 |  |  |  |  |
| **TXE** | 0.27 | 1.03 | 0.77 |  |  |  |  |

**Supplementary table 3:** Effect of endophytes treatments on tomato number of leaves per plant under glasshouse conditions by two-way ANOVA

| **TREATMENTS** | **NUMBER OF LEAVES PER PLANT** | | | | | | |
| --- | --- | --- | --- | --- | --- | --- | --- |
|  | **P42** | **HP3d** | **PGSS-1** | **A6** | ***P.fluorescens*** | **Control** | **Mean** |
| **Seed treatment** | 13.00 | 12.67 | 11.00 | 13.00 | 12.67 | 9.33 | 11.95 |
| **Seedling dip** | 19.33 | 18.33 | 18.67 | 18.00 | 19.33 | 8.67 | 17.06 |
| **Seed treatment +foliar spray** | 14.33 | 13.00 | 14.67 | 14.33 | 14.67 | 10.33 | 13.56 |
| **Seedling dip+ foliar spray** | 22.00 | 19.33 | 19.67 | 21.00 | 20.00 | 9.67 | 18.61 |
| **Seed treatment + seedling dip** | 16.00 | 15.33 | 16.67 | 18.33 | 17.33 | 9.33 | 15.50 |
| **Seed treatment + seedling dip+ foliar spray** | 24.67 | 20.67 | 21.33 | 23.00 | 24.33 | 8.67 | 20.45 |
| **Mean** | 18.22 | 16.56 | 17.00 | 17.94 | 18.06 | 9.33 |  |
|  | **SEm**$\boldsymbol{\pm}$ | **CD at 1%** | **CD at 5%** |  |  |  |  |
| **Treatments (T)** | 0.18 | 0.68 | 0.52 |  |  |  |  |
| **Endophytes (E)** | 0.18 | 0.68 | 0.52 |  |  |  |  |
| **TXE** | 0.45 | 1.68 | 1.26 |  |  |  |  |

**Supplementary table 4:** Effect of bacterial endophytes on % shoot dry matter of tomato under glasshouse conditions by two-way ANOVA

| **TREATMENTS** | **% SHOOT DRY MATTER** | | | | | | |
| --- | --- | --- | --- | --- | --- | --- | --- |
|  | **P42** | **HP3d** | **PGSS-1** | **A6** | ***P.fluorescens*** | **Control** | **Mean** |
| **Seed treatment** | 19.45  (26.17) | 18.37  (25.38) | 19.75  (26.38) | 19.66  (26.32) | 22.48  (28.30) | 18.18  (25.23) | 19.65  (26.30) |
| **Seedling dip** | 27.75  (31.79) | 28.99  (32.58) | 30.78  (33.70) | 29.27  (32.75) | 30.03  (33.23) | 18.99  (25.83) | 27.64  (31.65) |
| **Seed treatment +foliar spray** | 24.53  (29.69) | 22.22  (28.12) | 23.11  (28.73) | 23.11  (28.73) | 24.79  (29.86) | 18.96  (25.81) | 22.70  (28.49) |
| **Seedling dip+ foliar spray** | 28.98  (32.56) | 27.22  (31.44) | 31.17  (33.92) | 31.38  (34.05) | 33.40  (35.29) | 19.47  (26.16) | 28.61  (32.24) |
| **Seed treatment + seedling dip** | 24.29  (29.52) | 24.00  (29.33) | 25.09  (30.06) | 23.42  (28.94) | 25.85  (30.52) | 19.19  (25.97) | 23.64  (29.06) |
| **Seed treatment + seedling dip+ foliar spray** | 34.21  (35.79) | 29.96  (33.18) | 34.33  (35.86) | 35.11  (36.34) | 36.27  (37.03) | 18.23  (25.28) | 31.35  (33.91) |
| **Mean** | 26.54  (30.92) | 25.13  (30.00) | 27.37  (31.44) | 26.99  (31.19) | 28.81  (32.37) | 18.84  (25.71) |  |
|  | **SEm**$\boldsymbol{\pm}$ | **CD at 1%** | **CD at 5%** |  |  |  |  |
| **Treatments (T)** | 0.23 | 0.85 | 0.64 |  |  |  |  |
| **Endophytes (E)** | 0.23 | 0.85 | 0.64 |  |  |  |  |
| **TXE** | 0.56 | 2.08 | 1.57 |  |  |  |  |

*Values in parentheses are arcsine-transformed values

**Supplementary table 5:** Effect of bacterial endophytes on % root dry matter of tomato under glasshouse conditions by two-way ANOVA

| **TREATMENTS** | **% ROOT DRY MATTER** | | | | | | |
| --- | --- | --- | --- | --- | --- | --- | --- |
|  | **P42** | **HP3d** | **PGSS-1** | **A6** | ***P.fluorescens*** | **Control** | **Mean** |
| **Seed treatment** | 10.00  (18.42) | 9.70  (18.14) | 10.06  (18.48) | 10.15  (18.58) | 9.74  (18.19) | 8.43  (16.87) | 9.68  (18.11) |
| **Seedling dip** | 12.43  (20.64) | 12.44  (20.65) | 12.41  (20.63) | 12.30  (20.53) | 12.50  (20.71) | 8.75  (17.19) | 11.80  (20.06) |
| **Seed treatment +foliar spray** | 10.73  (19.12) | 10.71  (19.11) | 10.96  (19.34) | 10.87  (19.25) | 10.88  (19.25) | 7.82  (16.23) | 10.33  (18.72) |
| **Seedling dip+ foliar spray** | 12.71  (20.88) | 12.73  (20.90) | 12.96  (21.09) | 12.92  (21.07) | 12.96  (21.10) | 8.85  (17.28) | 12.19  (20.39) |
| **Seed treatment + seedling dip** | 11.87  (20.15) | 11.83  (20.11) | 11.86  (20.14) | 11.73  (20.03) | 11.68  (19.98) | 8.13  (16.57) | 11.18  (19.50) |
| **Seed treatment + seedling dip+ foliar spray** | 13.82  (21.83) | 13.40  (21.47) | 13.39  (21.46) | 13.65  (21.68) | 13.49  (21.55) | 7.83  (16.24) | 12.60  (20.71) |
| **Mean** | 11.93  (20.17) | 11.80  (20.06) | 11.94  (20.19) | 11.94  (20.19) | 11.86  (20.13) | 8.30  (16.73) |  |
|  | **SEm**$\boldsymbol{\pm}$ | **CD at 1%** | **CD at 5%** |  |  |  |  |
| **Treatments (T)** | 0.13 | 0.48 | 0.36 |  |  |  |  |
| **Endophytes (E)** | 0.13 | 0.48 | 0.36 |  |  |  |  |
| **TXE** | 0.31 | 1.18 | 0.89 |  |  |  |  |

*Values in parentheses are arcsine-transformed values

**Supplementary table 6:** Effect of endophytes treatments on size of lesion (l x w mm^2^) under glasshouse conditions by two-way ANOVA

| **TREATMENTS** | **LESION SIZE (mm^2^)** | | | | | | |
| --- | --- | --- | --- | --- | --- | --- | --- |
|  | **P42** | **HP3d** | **PGSS-1** | **A6** | ***P.fluorescens*** | **Control** | **Mean** |
| **Seed treatment** | 33.24 | 34.04 | 38.15 | 38.26 | 37.92 | 47.42 | 38.14 |
| **Seedling dip** | 5.03 | 4.13 | 4.15 | 6.27 | 5.46 | 48.19 | 12.21 |
| **Seed treatment +foliar spray** | 32.25 | 29.26 | 32.45 | 34.37 | \| 39.05 \| \| --- \| | 49.02 | 36.06 |
| **Seedling dip+ foliar spray** | 3.17 | 2.98 | 3.85 | 4.53 | 3.92 | 48.58 | 11.17 |
| **Seed treatment + seedling dip** | 10.65 | 10.05 | 12.61 | 13.92 | 10.88 | 48.45 | 17.76 |
| **Seed treatment + seedling dip+ foliar spray** | 2.55 | 1.89 | 2.74 | 2.76 | 1.74 | 48.76 | 10.07 |
| **Mean** | 14.48 | 13.73 | 15.66 | 16.69 | 16.49 | 57.46 |  |
|  | **SEm**$\boldsymbol{\pm}$ | **CD at 1%** | **CD at 5%** |  |  |  |  |
| **Treatments (T)** | 0.20 | 0.74 | 0.56 |  |  |  |  |
| **Endophytes (E)** | 0.20 | 0.74 | 0.56 |  |  |  |  |
| **TXE** | 0.49 | 1.82 | 1.37 |  |  |  |  |

**Supplementary table 7:** Effect of endophytes treatments on early blight severity (%) under glasshouse conditions by two-way ANOVA

| **TREATMENTS** | **DISEASE INTENSITY (%)** | | | | | | |
| --- | --- | --- | --- | --- | --- | --- | --- |
|  | **P42** | **HP3d** | **PGSS-1** | **A6** | ***P.fluorescens*** | **Control** | **Mean** |
| **Seed treatment** | 70.12 (56.88) | 71.81 (57.94) | 80.43 (63.75) | 80.73 (63.99) | 79.99  (63.44) | 100.0 (90.00) | 80.51  (66.00) |
| **Seedling dip** | 10.45 (18.86) | 8.58 (17.01) | 8.63 (17.05) | 12.99 (21.12) | 11.35  (19.68) | 100.0 (90.00) | 25.33  (30.62) |
| **Seed treatment +foliar spray** | 65.82 (54.23) | 59.69 (50.59) | 66.21 (54.46) | 70.12 (56.88) | 79.65  (63.19) | 100.00 (90.00) | 73.58  (61.56) |
| **Seedling dip+ foliar spray** | 6.54 (14.76) | 6.15 (14.34) | 7.94 (16.36) | 9.33  (17.78) | 8.07  (16.49) | 100.00 (90.00) | 23.01  (28.29) |
| **Seed treatment + seedling dip** | 22.04 (27.94) | 20.77 (27.10) | 26.05 (30.68) | 28.78 (32.43) | 22.45  (28.27) | 100.0 (90.00) | 36.68  (39.40) |
| **Seed treatment + seedling dip+ foliar spray** | 5.23 (13.22) | 3.87 (11.27) | 5.62 (13.70) | 5.66 (13.72) | 3.57  (10.87) | 100.00 (90.00) | 20.66  (25.46) |
| **Mean** | 30.03  (30.98) | 28.48  (29.71) | 32.48  (32.67) | 34.60  (34.32) | 34.18  (33.66) | 100.00  (90.00) |  |
|  | **SEm**$\boldsymbol{\pm}$ | **CD at 1%** | **CD at 5%** |  |  |  |  |
| **Treatments (T)** | 0.27 | 1.01 | 0.76 |  |  |  |  |
| **Endophytes (E)** | 0.27 | 1.01 | 0.76 |  |  |  |  |
| **TXE** | 0.66 | 2.49 | 1.87 |  |  |  |  |

*Values in parentheses are arcsine-transformed values

**Supplementary table 8:** Effect of different endophytes treatments on the plant growth parameters and early blight intensity of tomato under field conditions

| **Treatments** | **Plant ht.**  **(cm)** | **NBPP** | **NTPP** | **NFPT** | **NFPP** | **EDF**  **(mm)** | **AFW**  **(g)** | **FYPP**  **(Kg)** | **PDI** | **PDOC** |
| --- | --- | --- | --- | --- | --- | --- | --- | --- | --- | --- |
| \| **ST(P42)** \| \| --- \| | 63.80 | 4.50 | 10.00 | 2.00 | 20.50 | 56.50 | 90.30 | 0.90 | 38.0(38.0) | 5.00 |
| \| **SD(P42)** \| \| --- \| | 64.75 | 6.00 | 10.50 | 2.50 | 26.00 | 61.97 | 91.20 | 1.30 | 24.0(29.3) | 40.00 |
| \| **ST+SD(P42)** \| \| --- \| | 66.00 | 6.50 | 11.50 | 3.00 | 35.00 | 63.20 | 97.90 | 1.51 | 31.0(33.6) | 22.50 |
| \| **ST+SD+FS(P42)** \| \| --- \| | 71.80 | 7.00 | 13.00 | 3.50 | 41.00 | 64.90 | 102.1 | 1.60 | 24.0(29.3) | 40.00 |
| \| **ST(HP3d)** \| \| --- \| | 63.25 | 4.00 | 9.00 | 2.00 | 18.00 | 59.50 | 85.40 | 0.73 | 35.0(36.2) | 12.50 |
| \| **SD(HP3d)** \| \| --- \| | 64.15 | 4.00 | 9.50 | 2.00 | 18.00 | 60.17 | 91.90 | 0.75 | 27.5(31.6) | 31.30 |
| \| **ST+SD(HP3d)** \| \| --- \| | 66.55 | 4.50 | 10.50 | 2.50 | 25.50 | 63.70 | 92.80 | 1.28 | 27.0(31.3) | 32.50 |
| \| **ST+SD+FS(HP3d)** \| \| --- \| | 70.40 | 5.00 | 11.00 | 3.00 | 28.50 | 63.77 | 103.2 | 1.39 | 22.0(27.8) | 45.00 |
| \| **ST (PGSS 1)** \| \| --- \| | 67.10 | 5.00 | 9.50 | 2.50 | 20.50 | 54.83 | 64.80 | 0.95 | 35.0(36.2) | 12.50 |
| \| **SD (PGSS 1)** \| \| --- \| | 67.10 | 6.00 | 10.50 | 2.50 | 25.00 | 58.72 | 85.20 | 1.27 | 24.0(29.3) | 40.0 |
| \| **ST+SD (PGSS 1)** \| \| --- \| | 68.70 | 6.00 | 11.50 | 3.00 | 27.00 | 60.13 | 91.80 | 1.32 | 27.0(31.3) | 32.50 |
| \| **ST+SD+FS**  **(PGSS 1)** \| \| --- \| | 71.45 | 7.00 | 12.00 | 3.00 | 33.00 | 61.50 | 101.2 | 1.43 | 24.0(29.3) | 40.00 |
| \| **ST(A6)** \| \| --- \| | 64.60 | 5.00 | 10.00 | 2.50 | 24.00 | 56.17 | 81.30 | 1.22 | 38.0(38.0) | 5.00 |
| \| **SD(A6)** \| \| --- \| | 65.65 | 6.50 | 11.00 | 3.00 | 28.50 | 60.03 | 87.40 | 1.37 | 27.5(31.6) | 31.30 |
| \| **ST+SD(A6)** \| \| --- \| | 67.15 | 7.00 | 11.50 | 3.00 | 33.50 | 65.83 | 100.2 | 1.47 | 28.0(31.9) | 30.00 |
| \| **ST+SD+FS(A6)** \| \| --- \| | 67.40 | 8.00 | 13.50 | 3.00 | 38.50 | 67.00 | 102.2 | 1.55 | 27.5(31.6) | 31.30 |
| \| **ST (*P.fluorescens*)** \| \| --- \| | 71.75 | 5.00 | 9.50 | 2.50 | 20.50 | 56.50 | 83.80 | 1.00 | 32.0(34.4) | 20.00 |
| \| **SD (*P.fluorescens*)** \| \| --- \| | 68.60 | 6.50 | 11.00 | 3.00 | 30.00 | 58.50 | 92.20 | 1.41 | 26.0(30.6) | 35.00 |
| \| **ST+SD**  **(*P.fluorescens*)** \| \| --- \| | 70.80 | 6.50 | 11.00 | 3.00 | 28.50 | 62.23 | 100.5 | 1.38 | 26.0(30.2) | 35.00 |
| \| **ST+SD+FS**  **(*P.fluorescens*)** \| \| --- \| | 72.75 | 7.50 | 12.50 | 3.00 | 34.00 | 65.50 | 102.2 | 1.49 | 21.0(27.3) | 47.50 |
| \| **Captan (50% WP)** \| \| --- \| | 75.20 | 7.50 | 12.00 | 3.00 | 33.00 | 61.33 | 98.30 | 1.44 | 22.0(27.8) | 45.00 |
| \| **Control** \| \| --- \| | 61.00 | 4.00 | 9.00 | 2.00 | 16.50 | 54.83 | 64.20 | 0.85 | 40.0(39.2) | - |
| **SEm**$\boldsymbol{\pm}$ | 2.88 | 0.24 | 0.26 | 0.22 | 0.52 | 0.76 | 0.12 | 0.03 | 1.65 | - |
| **CD at 5%** | 8.47 | 0.71 | 0.78 | 0.63 | 1.52 | 2.17 | 0.36 | 0.10 | 4.85 | - |
| **CV%** | 7.37 | 7.10 | 4.20 | 13.82 | 3.25 | 2.17 | 0.23 | 4.59 | 8.91 | - |

NBPP-no. of branches per plant; NTPP-no. of trusses per plant; NFPT-no. of fruits per truss; NFPP-no. of fruits per plant; EDF-equatorial diameter of fruit; AFW-avg. fruit weight; FYPP-fruit yield per plant; PDI-Percent disease index; PDOC-Percent disease over control.
